# New Homes

**Published:** 1874-04

**Authors:** 


### NEW HOMES.

### MINNESOTA.

The State of Minnesota is located north of Iowa, and its western boundary is nearly midway between the Atlantic and Pacific Oceans. The altitude ranges from 600 feet to 1,400 feet above the level of the ocean.

The eastern portion of the State is heavily timbered. The forests are estimated at one sixth of the entire area. Their destruction is estimated at 270,000 acres a year. At this rate the entire State would be denuded of its forests in less than thirty years. The climate is severe in winter, but is not subject to those sudden changes which characterize States a few degrees south of it.

PRODUCTS.

Wheat is the staple product of Minnesota. In 1872 the number of acres sown was 1,267,309. The number of bushels produced was 22,059,375. Average per acre, bushels, 17.40.

OATS.

| | |
|---|---|
| Acres sown | 372,478 |
| Bushels produced | 12,550,733 |
| Average per acre, bushels | 33.69 |

CORN.

| | |
|---|---|
| Acres sown | 216,455 |
| Bushels produced | 7,142,245 |
| Average per acre, bushels | 32.99 |

BARLEY.

| | |
|---|---|
| Acres sown | 56,785 |
| Bushels produced | 1,495,494 |
| Average per acre, bushels | 26.33 |

RYE.

| | |
|---|---|
| Acres sown | 11,365 |
| Bushels produced | 182,730 |
| Average per acre, bushels | 16.07 |

BUCKWHEAT.

| | |
|---|---|
| Acres sown | 3,601 |
| Bushels produced | 49,359 |
| Average per acre, bushels | 13.70 |

POTATOES.

| | |
|---|---|
| Acres planted | 26,061 |
| Bushels produced | 3,072,349 |
| Average per acre, bushels | 117.89 |

HAY

| | |
|---|---|
| Acres cultivated | 88,990 |
| Tons produced | 108,028 |
| Average per acre, tons | 1.21 |
| Wild hay, tons | 743,414 |

APPLES.

| | |
|---|---|
| Apples produced, bushels | 39,663 |

WOOL.

| | |
|---|---|
| Number of sheep............................. | 125,723 |
| Pounds of wool sheared...................... | 497,045 |
| Average pounds per sheep................. . | 3.95 |

DAIRY PRODUCTS.

| | |
|---|---|
| Number of cows kept...................... | 135,691 |
| Pounds of butter produced................. | 8,823,660 |
| Pounds of cheese produced.................. | 772,630 |

TAXABLE PROPERTY.

| | |
|---|---|
| Number of acres assessed in 1873............. | 13,277,823 |
| Aggregate value............................ | 57,211,460 |
| Personal property......................... | 32,339,402 |

PUBLIC LANDS.

The area of the State is 80,784 square miles, or 51,701,760 acres. Number of acres surveyed up to August, 1873, was 34,-659,751. Amount disposed of by the United States, 23,668,956 acres. Balance not disposed of, 10,990,795 acres. Deducting pre-emption claims, the number of acres now surveyed and subject to entry at U. S. Land Offices is about 4,500,000. It is probable that the acreage yet subject to entry or purchase by individuals from the United States and from grantees, will be increased to at least 32,000,000 acres by the completion of the surveys. Of this total (excluding homesteads not patented to claimants) only 13,277,823 acres, divided into 58,000 farms, besides town and city lots, now contribute to the support of State and local government by the payment of taxes. Minnesota can still supply farms to one hundred thousand agriculturists from abroad. The present population of the State is 550,000.

METEOROLOGY.

In January, 1873, the coldest month of the year, the temperature ranged between 30 degrees above and 29 degrees below zero, a difference of 59 degrees, while the mean temperature for the month was 6.7 degrees above zero. The mean temperature for January, 1874, was 14 degrees above zero.

The barometer in January, 1873, showed a mean of 30.10; highest range, 30.75; lowest, 29.38; variation, 1.37. Relative humidity of the air in January, 67.9; February, 63.1; March, 64.

Amount of rain and melted snow, inches, 1.31 in January; 1.54 in February, and 1.34 in March, making 4.19 inches for the first three months out of a total of 33.74 for the year. This small deposit of moisture in winter, which is the rule in Minnesota, serves to give character to the winter climate of the State; and the air possesses a peculiar dryness, that enables a person to endure, without discomfort, a greater degree of cold than in States where the deposit of moisture is greater, and the atmosphere raw and damp.

